# Global variation in isolated posterior cruciate ligament reconstruction

**DOI:** 10.1186/s40634-022-00541-4

**Published:** 2022-10-09

**Authors:** Derrick M. Knapik, Varun Gopinatth, Garrett R. Jackson, Jorge Chahla, Matthew V. Smith, Matthew J. Matava, Robert H. Brophy

**Affiliations:** 1 Department of Orthopaedic Surgery, Division of Sports Medicine, Washington University in St. Louis, St. Louis, MO USA; 2 School of Medicine, Washington University in St. Louis, St. Louis, MO USA; 3 Saint Louis University, School of Medicine, St. Louis, MO USA; 4 Midwest Orthopaedics at Rush University, Chicago, IL USA

**Keywords:** Posterior cruciate ligament, Reconstruction, Knee, Regional variation

## Abstract

**Purpose:**

In the setting of persistent instability or failed non-operative management, surgical reconstruction is commonly recommended for isolated posterior cruciate ligament (PCL) tears. The purpose of this study was to systematically review published studies to evaluate regional variation in the epidemiology of and surgical approaches to primary, isolated PCL reconstruction.

**Methods:**

A systematic review was performed in June 2022 to identify studies examining operative techniques during primary, isolated PCL reconstruction. Collected variables consisted of reconstruction technique, graft type, graft source, tibial reconstruction technique, femoral and tibial drilling and fixation methods, and whether the remnant PCL was preserved or debrided. Studies were classified into four global regions: Asia, Europe, North America, and South America.

**Results:**

Forty-five studies, consisting of 1461 total patients, were identified. Most of the included studies were from Asia (69%, *n* = 31/45). Single bundle reconstruction was more commonly reported in studies out of Asia, Europe, and North America. Hamstring autografts were utilized in 51.7% (*n* = 611/1181) of patients from Asia and 60.8% (*n* = 124/204) of patients from Europe. Trans-tibial drilling and outside-in femoral drilling were commonly reported in all global regions. The PCL remnant was generally debrided, while remnant preservation was commonly reported in studies from Asia.

**Conclusion:**

Surgical treatment of isolated PCL injuries varies by region, with the majority of published studies coming from Asia. Single-bundle reconstruction with hamstring autograft through a trans-tibial approach is the most commonly reported technique in the literature, with males reported to undergo isolated reconstruction more often than females.

**Level of Evidence:**

Systematic review, Level IV.

**Supplementary Information:**

The online version contains supplementary material available at 10.1186/s40634-022-00541-4.

## Introduction

Injuries to the posterior cruciate ligament (PCL) have been reported with increasing frequency, accounting for up to 17% of all knee injuries [43]. The estimated annual incidence of isolated PCL injuries has been reported as 2 per 100,000 persons [57]. Injuries to the PCL have also become increasingly recognized as a common cause of morbidity and limited knee function [11], increasing the risk for the development of degenerative changes and reduced joint longevity [32, 57]. While an improved understanding of the anatomy and biomechanical function of the PCL has emerged in recent years [23, 27, 28, 51, 54, 73], along with advancements in surgical techniques and instrumentation for the treatment of PCL injuries [6], PCL reconstruction remains a complex and challenging surgical procedure. This complexity is compounded by a lack of familiarity with the operation due to the relatively low number of patients requiring the procedure, as well as the proximity of the neurovascular bundle [46].

While traditionally managed non-operatively, [16, 74] operative indications for isolated PCL injuries have expanded to include complete (grade III) injuries, and patients with grade II injuries with residual posterior laxity and disability following non-operative management in a structured rehabilitation program [50, 51, 61, 75]. Surgical reconstruction for isolated injuries has become increasingly performed, with biomechanical studies demonstrating greater sagittal and rotational translation in the PCL-deficient knee, resulting in higher patellofemoral and medial tibiofemoral contact pressures and the potential for damage within the joint [21, 36]. Two systematic reviews evaluating 27 (*n* = 5197 patients) [59] and 23 studies (*n* = 781 patients) [5] reported that patients undergoing surgical management for isolated PCL injuries possessed a greater reduction in posterior laxity when compared to patients treated non-operatively.

Despite the increased popularity of operative management for isolated PCL injuries, a variety of surgical reconstruction techniques and approaches have been reported [19]. Common variables include graft type and source, bundle number, femoral and tibial drilling technique, method of tibial and femoral graft fixation, and preservation versus debridement of the remnant PCL [34, 42, 53, 73]. To date, no investigation has evaluated the potential for global differences in isolated PCL reconstruction techniques. Geographic differences in any surgical technique can be attributed to a variety of causes reflective of differences in surgical training, personal experience, practice focus, cultural mores, and religious beliefs. Therefore, the purpose of this study was to systematically review the orthopedic literature to assess for regional variation in the epidemiology of and surgical approaches to primary, isolated PCL reconstruction. The authors hypothesized that regional differences would be present based on reconstruction technique and graft choice.

## Methods

A systematic review was conducted according to the 2020 Preferred Reporting Items for Systematic Reviews and Meta-Analyses (PRISMA) statement [52]. Following registration on the PROSPERO International Prospective Register of Systematic Reviews (*ID # 279879*), a literature search identifying studies evaluating outcomes following isolated PCL reconstruction from January 1995 to May 2022 was performed on June 21, 2022 using the following databases: PubMed, EMBASE, OVID, Scopus and the Cochrane Library. Each search included a variable combination of the following terms: ‘posterior cruciate ligament’ OR ‘reconstruction’, OR ‘isolated’ OR ‘knee’ OR ‘surgery’ OR ‘region’ OR ‘single-bundle’ OR ‘double bundle’ OR ‘transtibial’ OR ‘tibial inlay’ OR ‘tunnel’ OR ‘femur’ OR ‘tibia’ OR ‘fixation’ OR ‘graft’ AND ‘outcome measure’.

The inclusion criteria consisted of studies published in the English language or with English-language translation, reporting operative techniques and approaches for the primary treatment of isolated PCL injuries. Exclusion criteria consisted of: studies reporting the results of PCL surgery in the setting of multi-ligament knee injuries involving the anterior cruciate ligament, medial or lateral collateral ligament, primary PCL repair, biomechanical, anatomic or animal studies, epidemiological and national database studies, editorial articles, review articles, and systematic reviews/meta-analyses. Studies reporting on patients undergoing revision PCL reconstruction were also excluded, along with studies that failed to adequately describe surgical methods. Studies with overlapping patient data were considered separately with inclusion of those investigations reporting the most recent follow-up.

Two authors [D.M.K., V.G.] independently performed the initial search by screening articles in the following systematic approach: assessment of duplicate articles, content within the article title, content of the abstract, and full-text review. Any disagreements in study selection were discussed and decided by a third independent author R.H.B. To confirm that no studies were missing from the systematic review, all references cited in the included studies were also reviewed and reconciled.

Studies were classified into four global regions based on the primary investigative site: Asia, Europe, North America, and South America. Mean patient age and sex were recorded from each study. Reported injury mechanism(s) and graft source were analyzed based on the number of patients reported. Reconstruction technique (single- versus double-bundle), graft source (autograft versus allograft), tibial reconstruction technique (trans-tibial versus tibial inlay), femoral drilling method (outside-in versus inside-out), tibial fixation method, femoral fixation method, and whether the remnant PCL was preserved or debrided were analyzed based on the number of studies in which each variable was reported. When multiple surgical techniques or grafts were reported in the same study, each variable was separately recorded in its respective category. Patient reported outcome measures (PROMs) were recorded, when reported.

A methodological quality assessment of the included studies was performed by two authors (initials blinded for peer review) to ensure bias was minimized using the Newcastle–Ottawa Scale (NOS) for studies of level I-III evidence (Table 1) and the National Institute of Health (NIH) Quality Assessment for level IV evidence studies (Table 2). For each region, patient demographics, variation across surgical techniques, and proportion of studies reporting the most common patient-reported outcome measures (PROMs) were calculated and analyzed. Continuous variables were presented as means and standard deviations, while categorical variables were presented as percentages. Statistical analyses were performed using Microsoft Excel (V. 16.63.1, Microsoft, Redmond, WA, United States).

**Table 1:** Newcastle–Ottawa Scale (NOS) quality assessment of the included studies. Each study was evaluated on points: the selection of study groups; the comparability of the groups; and the ascertainment of the outcomes measured. A star indicates that the study met the requirements. Each study can be awarded a maximum of nine stars

**Newcastle–Ottawa Quality Scale** ★												
	**Selection**				**Comparability**			**Outcome**				
Study (Year)	Representativeness of treated cohort	Selection of comparative cohort	Ascertainment of treated cohort records	Outcome of interest was not present at start	Controls for age/sex	Controls for any additional factor	Assessment of outcome		Long enough follow-up	Adequacy of follow-up	Total Quality Score	
Chen et al. (2002) [14]	★	★	★	★	★	★	★		★	★	9	
Lee et al. (2013) [33]	★	★	★	★	0	0	★		★	★	7	
Li et al. (2014) [37]	★	★	★	★	★	★	★		★	★	9	
Li et al. (2015) [35]	★	★	★	★	★	★	★		★	★	9	
Lin et al. (2013) [40]	★	★	★	★	★	★	★		★	★	9	
MacGillivray et al. (2006) [42]	★	★	★	★	★	★	★		★	★	9	
Ochiai et al. (2019) [49]	★	0	★	★	0	0	★		★	★	6	
Rhatomy et al. (2021) [56]	★	★	★	★	★	★	★		★	★	9	
Saragaglia et al. (2020) [58]	★	★	★	★	★	★	★		★	★	9	
Seon et al. (2006) [63]	★	★	★	★	0	0	★		★	★	7	
Song et al. (2014) [66]	★	★	★	★	★	★	★		★	★	9	
Tachibana et al. (2021) [69]	★	★	★	★	★	★	★		★	★	9	
Wang et al. (2004) [72]	★	★	★	★	0	★	★		★	★	8	
Wong et al. (2009) [76]	★	★	★	★	0	★	★		★	★	8	
Xu et al. (2014) [78]	★	★	★	★	★	★	★		★	★	9	
Yang et al. (2012) [79]	★	★	★	★	★	★	★		★	★	9	
Yoon et al. (2019) [80]	★	★	★	★	0	★	★		★	★	8	
Zhao et al. (2007) [83]	★	★	★	★	0	★	★		★	★	8	
Zhao et al. (2009) [85]	★	0	★	★	0	0	★		★	★	6

**Table 2:** The National Institute of Health (NIH) Quality Assessment Tool quality assessment. Study quality was rated as 0 for poor (0–4 out of 14 questions), i for fair (5–10 out of 14 questions), or ii for good (11–14 out of 14 questions)

**NIH Quality Assessment** ✔															
Study (Year)	Was the research question or objective in this paper clearly stated?	Was the study population clearly specified and defined?	Was the participation rate of eligible persons at least 50%?	Were all the subjects selected or recruited from the same or similar populations?	Was a sample size justification, power description, or variance and effect estimates provided?	For the analyses in this paper, were the exposure(s) of interest measured prior to the outcome(s) being measured?	Was the timeframe sufficient so that one could reasonably expect to see an association between exposure and outcome if it existed?	For exposures that can vary in amount or level, did the study examine different levels of the exposure?	Were the exposure measures (independent variables) clearly defined, valid, reliable, and implemented consistently across all study participants?	Was the exposure(s) assessed more than once over time?	Were the outcome measures (dependent variables) clearly defined, valid, reliable, and implemented? consistently across all study participants?	Were the outcome assessors blinded to the exposure status of participants?	Was loss to follow-up after baseline 20% or less?	Were key potential confounding variables measured and adjusted statistically for their impact on the relationship? between exposure(s) and outcome(s)?	Summary Quality
Adachi et al. (2007) [1]	✔	✔	✔	✔	X	✔	✔	X	X	✔	✔	✔	✔	X	i
Ahn et al. (2006) [4]	✔	✔	✔	✔	X	✔	✔	X	✔	X	✔	✔	✔	X	i
Ahn et al. (2013) [3]	✔	✔	✔	✔	✔	✔	✔	X	✔	X	✔	✔	✔	X	ii
Boutefnouchet et al. (2013) [9]	✔	✔	✔	✔	X	X	✔	X	✔	X	✔	✔	✔	X	i
Chan et al. (2006) [12]	✔	✔	✔	✔	X	✔	✔	X	✔	X	✔	X	✔	X	i
Chen et al. (2009) [13]	✔	✔	✔	✔	X	✔	✔	X	✔	X	✔	X	✔	X	i
Chen et al. (2012) [15]	✔	✔	✔	✔	X	✔	✔	X	✔	X	✔	X	✔	X	i
Cury et al. (2012) [17]	✔	✔	✔	✔	X	✔	✔	✔	✔	X	✔	X	✔	X	i
Eguchi et al. (2014) [18]	✔	✔	✔	✔	X	✔	✔	X	✔	✔	✔	X	✔	X	i
Garofalo et al. (2006) [20]	✔	✔	X	✔	X	✔	✔	✔	✔	X	✔	X	✔	X	i
Gill et al. (2009) [22]	✔	✔	✔	✔	X	✔	✔	X	✔	X	✔	X	✔	X	i
Hermans et al. (2009) [24]	✔	✔	✔	✔	X	X	✔	X	✔	X	✔	✔	✔	X	i
Ihle et al. (2014) [25]	✔	✔	X	✔	X	X	✔	✔	✔	X	✔	X	✔	X	i
Jung et al. (2004) [26]	✔	✔	✔	✔	X	✔	✔	X	✔	X	✔	X	✔	X	i
Lahner et al. (2012) [31]	✔	✔	X	✔	X	✔	✔	✔	✔	X	✔	X	✔	X	i
Lien et al. (2010) [38]	✔	✔	✔	✔	X	✔	✔	X	✔	X	✔	✔	✔	X	i
Lim et al. (2010) [39]	✔	✔	✔	✔	X	✔	✔	X	✔	X	✔	✔	✔	X	i
Mariani et al. (1997) [44]	✔	✔	✔	✔	X	✔	✔	✔	✔	X	✔	X	✔	X	i
Noh et al. (2017) [47]	✔	✔	✔	✔	✔	✔	✔	✔	✔	X	✔	X	✔	X	ii
Norbakhsh et al. (2014) [48]	✔	✔	✔	✔	X	✔	✔	✔	✔	X	✔	X	✔	X	i
Rauck et al. (2019) [55]	✔	✔	✔	✔	X	✔	✔	✔	✔	X	✔	X	✔	X	i
Sekiya et al. (2005) [62]	✔	✔	✔	✔	X	✔	✔	✔	✔	X	✔	✔	✔	X	ii
Shon et al. (2010) [65]	✔	✔	✔	✔	X	✔	✔	✔	✔	X	✔	X	✔	✔	ii
Wu et al. (2007) [77]	✔	✔	✔	✔	X	✔	✔	✔	✔	X	✔	X	✔	X	i
Zayni et al. (2011) [82]	✔	✔	✔	✔	X	✔	✔	X	✔	X	✔	X	✔	X	i
Zhao (2008) [84]	✔	✔	✔	✔	X	✔	✔	✔	✔	✔	✔	X	✔	X	ii

## Results

**Fig. 1:**
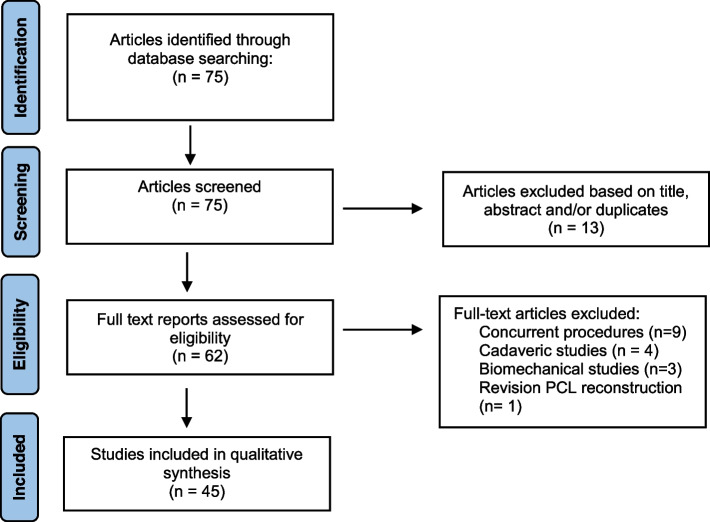
Preferred Reporting Items for Systematic reviews and Meta-Analyses (PRISMA) flowchart of study

Following the literature review, a total of 75 articles were identified. No disagreements between the two authors were encountered. The search process is outlined in the flow diagram (Fig. 1). During title and abstract assessment, a total of 62 studies were selected for full-text evaluation. Following full-text evaluation, a total of 45 articles meeting inclusion/exclusion criteria were identified, consisting of 1461 total patients.

Studies from Asia [1, 3, 4, 12–15, 18, 26, 33, 35, 37, 39, 40, 47–49, 56, 63, 65, 66, 69, 72, 76–80, 83–85] (69%, *n* = 31/45) comprised the majority of included articles, followed by Europe [9, 20, 24, 25, 31, 38, 44, 58, 82] (20%, *n* = 9/45), North America [22, 42, 55, 62] (8.9%, *n* = 4/45), and South America [17] (2.2%, *n* = 1/45)  (Table 3, Additional file 1: Table S1). Males comprised 77% (*n* = 1120/1461) of patients. Motor vehicle accidents were the most common injury mechanism in patients reported from studies out of Asia (55%, *n* = 453/827) and South America (64%, *n* = 9/14), while sports-related injuries represented the most common mechanisms of injury in patients from Europe (52%, *n* = 70/135) and North America (56%, *n* = 19/34). Single-bundle PCL reconstruction was reported in a higher number of studies from Asia (77%, *n* = 24/31), Europe (78%, *n* = 7/9) and North America (100%, *n* = 4/4). Autografts were the most commonly utilized graft in patients reported in studies from Asia (66.7%, *n* = 788/1181), Europe (96.6%, *n* = 197/204), and South America (100%, *n* = 14/14), while 69.4% (*n* = 43/62) of patients from North America underwent isolated PCL reconstruction with allografts. Hamstring autografts were utilized in 51.7% (*n* = 611/1181) of patients from studies out of Asia, 60.8% of patients from Europe (*n* = 124/204), and 100% of patients from South America (*n* = 14/14). The most common graft reported in patients from North America was the Achilles tendon allograft (58.1%, *n* = 36/62). Use of the Ligament Advanced Reinforcement System (LARS) synthetic graft was reported in studies from Asia (4.8%, *n* = 57/1181) and Europe (3.9%, *n* = 8/204).

**Table 3:** Overview of study variables during isolated posterior cruciate ligament reconstruction based on global region

	Asia (*n* = 31)	Europe (*n* = 9)	N. America (*n* = 4)	S. America (*n* = 1)
Age	30.7 +—3.7	28.4 +—2.31	32.6 +—4.8	31.0 +—0
Gender (% male/total patients)	.772 (912/1181)	.765 (156/204)	.694 (43/62)	.643 (9/14)
Injury Mechanism (*n* = # patients reported)				
Motor Vehicle Accident	.548 (453/827)	.429 (58/135)	.294 (10/34)	.643 (9/14)
Sports	.320 (265/827)	.519 (70/135)	.559 (19/34)	.357 (5/14)
Falls	.047 (39/827)	0 (0/135)	.112 (4/34)	0 (0/14)
Other	.084 (70/827)	.052 (7/135)	.029 (1/34)	0 (0/14)
Bundle Number (*n* = # of studies reporting)				
Single	0.774 (24/31)	0.778 (7/9)	1.000(4/4)	0 (0/1)
Double	0.258 (8/31)	0.333 (3/9)	0.250 (1/4)	1 (1/1)
Graft Source (*n* = # of patients reported)				
Hamstring autograft	.517 (611/1181)	.608 (124/204)	.032 (2/62)	1 (14/14)
Achilles tendon allograft	.203 (240/1181)	.044 (9/204)	.581 (36/62)	0 (0/14)
Quadriceps tendon autograft	.051 (60/1181)	.103 (21/204)	.113 (7/62)	1 (14/14)
Tibialis Anterior allograft	.107 (126/1181)	0 (0/204)	0 (0/62)	0 (0/14)
Bone-patellar-tendon-bone autograft	.054 (64/1181)	.328 (67/204)	.177 (11/62)	0 (0/14)
Bone-patellar-tendon-bone allograft	.008 (10/1181)	0 (0/204)	.113 (7/62)	0 (0/14)
Other	.045 (53/1181)	0 (0/204)	0 (0/62)	0 (0/14)
Autograft	.667 (788/1181)	.966 (197/204)	.322 (20/62)	1 (14/14)
Allograft	.318 (376/1181)	.147 (30/204)	.694 (43/62)	0 (0/14)
Artificial Graft (LARS)	.048 (57/1181)	.039 (8/204)	0 (0/62)	0 (0/14)
Tibial Technique (*n* = # of studies reporting)				
Transtibial	.935 (29/31)	1 (9/9)	1 (4/4)	1 (1/1)
Tibial Inlay	.129 (4/31)	0 (0/9)	.5 (2/4)	0 (0/1)
Femoral Tunnel Drilling (*n* = # of studies reporting)				
Outside-In	.462 (12/26)	.571 (4/7)	1 (3/3)	1 (1/1)
Inside-Out	.538 (14/26)	.429 (3/7)	0 (0/3)	0 (0/1)
Tibial Fixation (*n* = # of studies reporting)				
Screw	.645 (20/31)	.875 (7/8)	1 (2/2)	1 (1/1)
Spike/Staple	.129 (4/31)	.125 (1/8)	0 (0/2)	0 (0/1)
Button	.097 (3/31)	0 (0/8)	0 (0/2)	0 (0/1)
Other	0 (0/31)	0 (0/8)	0 (0/2)	0 (0/1)
Femoral Fixation (*n* = # of studies reporting)				
Screw	.581 (18/31)	.875(7/8)	1 (3/3)	1 (1/1)
Button	.226 (7/31)	.125(1/8)	0 (0/3)	0 (0/1)
Mini-Plate	.097 (3/31)	0(0/8)	0 (0/3)	0 (0/1)
Other	.097 (3/31)	0(0/8)	0 (0/3)	0 (0/1)
Remnant (*n* = # studies reporting)				
Preserved	0.864 (19/22)	.2 (1/5)	0 (0/2)	0 (0/1)
Debrided	.182 (4/22)	.8 (4/5)	1 (2/2)	1 (1/1)
Reported PROMs (*n* = # studies reporting)				
Lysholm	.903 (28/31)	.889 (8/9)	.5 (2/4)	1 (1/1)
Tegner	.613 (19/31)	1 (9/9)	.5 (2/4)	0 (0/1)
IKDC	.774 (24/31)	1 (9/9)	.75 (3/4)	1 (1/1)
Posterior Drawer	.419 (13/31)	.444 (4/9)	.5 (2/4)	1 (1/1)

*Legend*: *LARS* Ligament Advanced Reinforcement System, *PROM* Patient reported outcomes measures, *IKDC* International Knee Documentation Committee

Across all regions, the trans-tibial technique was most commonly utilized when compared to the tibial inlay technique (Table 3, Additional file 1: Table S1). In Europe, North America, and South America, 100% (*n* = 14/14) of studies utilized the trans-tibial technique, compared to 93.5% (*n* = 29/31) of studies in Asia. Femoral tunnel drilling with an outside-in technique was reported in 54.1% (*n* = 20/37) of all studies. Tibial graft fixation primarily involved interference screws (71%, *n* = 30/42), followed by spike/staple fixation (12%, *n* = 5/42) and buttons (7%, *n* = 3/42). Interference screw fixation was the most commonly reported method of femoral fixation (67%, *n* = 29/43), followed by button fixation (19%, *n* = 8/43) and mini-plates (7%, *n* = 3/43). Debridement of the PCL remnant was performed commonly in studies from Europe (80%, *n* = 4/5), North America (100%, *n* = 2/2), and South America (100%, *n* = 1/1), while remnant preservation was reported in 86.4% (*n* = 19/22) of studies from Asia.

Lysholm knee score, Tegner activity level scale, and International Knee Documentation Committee (IKDC) score were the most commonly reported PROMs across all regions (Additional file 2: Table S2). Posterior drawer grades were similarly reported in studies across all regions. Additional outcome measures, including the Western Ontario and McMaster University Osteoarthritis Index (WOMAC), Cincinnati Knee rating system, and Hospital for Special Surgery (HSS) score were less commonly reported. There was infrequent reporting of absolute posterior tibial translation and/or side-to-side differences. Complications were reported in 53.3% (*n* = 24/45) of studies comprising a total of 179 patients. The most commonly reported complications consisted of discomfort with kneeling (8.9%, *n* = 16/179 patients), paresthesia (8.4%, *n* = 15/179 patients), crepitus (6.7%, *n* = 12/179 patients), symptomatic hardware requiring removal (6.1%, *n* = 11/179 patients), infection (5.0%, *n* = 9/179 patients), and decreased range of motion (2.8%, *n* = 5/179 patients).

## Discussion

The most important findings of this investigation were that the majority of studies of patients undergoing isolated PCL reconstruction were reported from Asia. Isolated PCL reconstruction was performed primarily in males in all regions, most often using a single-bundle hamstring autograft through a trans-tibial tunnel with interference screw fixation on the tibia and femur. Debridement of the remnant PCL was performed in all regions except in Asia where it was preserved in most studies.

Across all four global regions, males underwent isolated PCL reconstruction more commonly than females, which is in agreement with prior investigations [32, 60]. Specifically, LaPrade et al. [32] observed that PCL injuries were more prevalent in males, whether isolated or combined with other injuries when compared to females. This finding may be related to the higher rate of male participation in contact sports, such as Association football and cricket in Asia, American football and basketball in North America, and soccer in Europe and South America. Participation in these activities, especially at a high level, may require surgical reconstruction, even in the presence of isolated PCL injuries, to restore stability and allow successful and effective return to sport [68]. Meanwhile, other traumatic mechanisms of injury, such as motor vehicle injuries and falls remain a potential etiology behind PCL injuries [20, 38]. There remains limited evidence regarding the influence of anatomic differences between males and females sustaining PCL injuries. Van Kujik et al. [71] reported on radiographic measures of intercondylar notch width and shape in 94 patients with PCL rupture compared to 168 age and sex-matched controls. The authors observed that patients with PCL injuries possessed a smaller and more sharply angled notch. However, no separate analyses evaluating differences between male and female patients was performed. Meanwhile, Liu et al. [41] found in their investigation analyzing 103 patients (*n* = 41 females; *n* = 62 males) with PCL ruptures compared to age and sex-matched controls, that the greatest risk factor for PCL injury was a greater coronal notch width in females and decreased coronal condylar width in males. Further studies identifying specific epidemiologic and anatomic factors contributing to the higher reported prevalence of isolated PCL reconstruction in males are warranted.

Single-bundle PCL reconstruction was the most common graft configuration reported in the majority of studies. The relative worth of single- versus double-bundle reconstruction continues to be debated in the current literature [31]. Biomechanical investigations have demonstrated that the anterolateral and posteromedial bundles each resist posterior tibial translational at different degrees of knee flexion, supporting the concept of codominant, synergistic roles for the two PCL bundles [2, 27]. Traditional single-bundle reconstruction techniques have been shown to primarily restore the anterolateral bundle [51, 73]. Wijdicks et al. [73] observed in their biomechanical study that the double-bundle reconstruction effectively enabled restoration of near normal knee kinematics with improved rotational stability when compared to the single-bundle reconstruction. Several clinical investigations have similarly observed improved restoration of native knee anatomy and kinematics using a double-bundle reconstruction [6, 24, 29, 54]. Kim et al. [30] reported that single-bundle PCL reconstruction improved posterior knee laxity, as well as clinical outcome scores based on Lysholm and IKDC scores; however, restoration of native knee stability was not re-established. Similarly, Li et al. [37] reported improved side-to-side differences in posterior tibial translation in patients undergoing double-bundle PCL reconstruction (2.2 mm) when compared to single-bundle reconstruction (4.1 mm). A systematic review and meta-analysis comparing single- and double-bundle PCL reconstructions by Chahla et al. [10] observed significant improvement in posterior tibial translation and objective IKDC scores in patients undergoing double-bundle reconstruction without significant differences in postoperative Lysholm or Tegner scores. Despite the concern for persistent posterior laxity with single-bundle reconstruction, this technique is still more commonly reported, especially in studies performed in Asia. The specific reasoning behind this finding, whether secondary to cost, operative time, or the complexity associated with the double-bundle technique, warrants further investigation.

The use of autograft tissue, with the most common source being the hamstrings, represented the most frequently reported graft type. Clinical studies comparing autograft and allograft PCL reconstruction are limited. Sun et al. [67] reported that despite comparable functional scores following PCL reconstruction, patients treated with autografts possessed improved posterior knee stability when compared to allografts. Li et al. [35] similarly observed equivalent outcomes in patients undergoing single-bundle PCL reconstruction utilizing hamstring tendon autografts versus tibialis anterior allografts. Wang et al. [72] reported that despite comparable outcomes in patients reconstructed with autograft versus allograft tissue, there was an increased risk of complications with autografts related primarily to infection and donor-site morbidity. A meta-analysis of five studies (1 randomized controlled trial [RCT], 4 non-RCTs) performed by Tian et al. [70] reported no significant differences in Lysholm, IKDC, or posterior stability in patients reconstructed with autograft tendons compared to those treated with allografts (*p* = 0.04). However, the authors concluded that there is currently insufficient evidence to determine the superiority of one graft type over the other. Therefore, the optimal graft source for isolated PCL reconstruction remains controversial, with the frequent use of autograft across all global regions potentially related to limited allograft availability, high costs, surgeon bias, or concerns for disease transmission or graft rejection [70].

Trans-tibial drilling was more commonly reported when compared to a tibial inlay technique. While initially designed to avoid the sharp angle (“killer curve”) present at the proximal aperture of the tibial tunnel, potentially leading to graft damage and failure [7, 8], it is likely that the necessity of performing an open posterior approach, increasing risk of injury to the saphenous nerve or popliteal neurovascular structures, may be responsible for the infrequent performance of the open tibial inlay technique [81]. In addition, the risk of nonunion of the inlay bone plug, along with the development of popliteal adhesions to the posterior capsule complicating revisions procedures, may account for the popularity of trans-tibial drilling [32]. The recent evolution of an all-arthroscopic inlay technique that avoids the posterior exposure may increase the popularity of the inlay method in that it not only avoids the “killer curve” associated with a trans-tibial tunnel, but also some neurovascular risks associated with the open inlay procedure.

Significant differences in either biomechanical or clinical outcomes between the trans-tibial and tibial inlay techniques have not been reported. McAllister et al. [45] found no significant differences in mean knee laxities between the tibial tunnel and tibial inlay techniques at any knee flexion angle; as both reconstruction techniques restored mean knee posterior laxity to within 1.6 mm of the intact knee values over the entire range of knee motion. Shin et al. [64] observed no significant differences in their systematic review of 7 studies comparing outcomes in patients undergoing trans-tibial drilling versus either open or arthroscopic tibial inlay during single-bundle PCL reconstruction. Meanwhile, Song et al. [66] found similar clinical and radiographic outcomes between the two techniques in 66 patients after a mean follow-up of 148 months. Therefore, despite comparable outcomes, the potential risks and complexity associated with the open tibial inlay procedure have likely tempered surgeon interest in this procedure. Further studies comparing the trans-tibial and arthroscopic inlay techniques are warranted.

This study is not without limitations. Due to the heterogeneity of reported surgical techniques in the four global regions examined, we could not perform any direct comparison of outcomes based on the techniques utilized in each region. Moreover, due to the small number of studies meeting our inclusion criteria, especially in the North and South American regions, the performance of any meaningful statistical analyses was limited. Evaluation of outcomes based on posterior laxity was not performed due to the significant variation of techniques used to assess posterior tibial laxity, including KT-1000 arthrometer and various stress radiographic techniques [51]. The incidence and severity of osteoarthritic development were not analyzed due to the large variation in reported follow-up. The higher prevalence of isolated PCL reconstructions performed in a certain global region does not imply that the procedure and techniques are preferentially performed by the majority of surgeons in that region. This study was only able to evaluate the published literature, which was generated predominantly by academic centers. Unfortunately, there are no comprehensive international repositories of surgical data that would be required to definitively determine the procedures and techniques that are actually being performed by all surgeons in each global region. Despite this study being limited to patients sustaining isolated PCL injuries, the true prevalence of concurrent meniscal and chondral injuries requiring intervention that were not reported or overlooked during intervention, cannot be inferred, leading to a potential bias in interpreting the data. We did not include surgical treatment of multi-ligamentous knee injuries, which may be the most common indication for PCL reconstruction. Lastly, due to the infrequent reporting of injury grade, this variable was not accounted for in our analysis.

## Conclusions

Surgical treatment of isolated PCL injuries varies by region, with the majority of published studies coming from Asia. Single-bundle reconstruction with hamstring autograft through a trans-tibial approach is the most commonly reported technique in the literature, with males reported to undergo isolated reconstruction more often than females.

## Supplementary Information

**Additional file 1: Table S1.** Overview of included studies based on global region.

**Additional file 2: Table S2.** Outcomes following isolated PCL reconstruction.

## References

[1] Adachi N, Ochi M, Uchio Y, Iwasa J, Ishikawa M, Shinomiya R (2007). Temporal change of joint position sense after posterior cruciate ligament reconstruction using multi-stranded hamstring tendons. Knee Surg Sports Traumatol Arthrosc.

[2] Ahmad CS, Cohen ZA, Levine WN, Gardner TR, Ateshian GA, Mow VC (2003). Codominance of the individual posterior cruciate ligament bundles. An analysis of bundle lengths and orientation. Am J Sports Med.

[3] Ahn JH, Lee YS, Choi SH, Chang MJ, Lee DK (2013). Single-bundle transtibial posterior cruciate ligament reconstruction using a bioabsorbable cross-pin tibial back side fixation. Knee Surg Sports Traumatol Arthrosc.

[4] Ahn JH, Yang HS, Jeong WK, Koh KH (2006). Arthroscopic transtibial posterior cruciate ligament reconstruction with preservation of posterior cruciate ligament fibers: clinical results of minimum 2-year follow-up. Am J Sports Med.

[5] Ahn S, Lee YS, Song YD, Chang CB, Kang SB, Choi YS (2016). Does surgical reconstruction produce better stability than conservative treatment in the isolated PCL injuries?. Arch Orthop Trauma Surg.

[6] Anderson CJ, Ziegler CG, Wijdicks CA, Engebretsen L, LaPrade RF (2012). Arthroscopically pertinent anatomy of the anterolateral and posteromedial bundles of the posterior cruciate ligament. J Bone Joint Surg Am.

[7] Berg EE (1995). Posterior cruciate ligament tibial inlay reconstruction. Arthroscopy.

[8] Bergfeld JA, McAllister DR, Parker RD, Valdevit AD, Kambic HE (2001). A biomechanical comparison of posterior cruciate ligament reconstruction techniques. Am J Sports Med.

[9] Boutefnouchet T, Bentayeb M, Qadri Q, Ali S (2013). Long-term outcomes following single-bundle transtibial arthroscopic posterior cruciate ligament reconstruction. Int Orthop.

[10] Chahla J, Moatshe G, Cinque ME (2017). Single-Bundle and Double-Bundle Posterior Cruciate Ligament Reconstructions: A Systematic Review and Meta-analysis of 441 Patients at a Minimum 2 Years' Follow-up. Arthroscopy.

[11] Chahla J, von Bormann R, Engebretsen L, LaPrade RF (2016). Anatomic posterior cruciate ligament reconstruction: State of the Art. Journal of ISAKOS.

[12] Chan YS, Yang SC, Chang CH (2006). Arthroscopic reconstruction of the posterior cruciate ligament with use of a quadruple hamstring tendon graft with 3- to 5-year follow-up. Arthroscopy.

[13] Chen B, Gao S (2009). Double-bundle posterior cruciate ligament reconstruction using a non-hardware suspension fixation technique and 8 strands of autogenous hamstring tendons. Arthroscopy.

[14] Chen CH, Chen WJ, Shih CH (2002). Arthroscopic reconstruction of the posterior cruciate ligament: a comparison of quadriceps tendon autograft and quadruple hamstring tendon graft. Arthroscopy.

[15] Chen CP, Lin YM, Chiu YC (2012). Outcomes of arthroscopic double-bundle PCL reconstruction using the LARS artificial ligament. Orthopedics.

[16] Cosgarea AJ, Jay PR (2001). Posterior cruciate ligament injuries: evaluation and management. J Am Acad Orthop Surg.

[17] Cury RdPL, Severino NR, Camargo OPA, Aihara T, de Oliveira VM, Avakian R (2012). Posterior Cruciate Ligament Reconstruction with Autograft of the Double Semitendinosus Muscles and Middle Third of the Quadriceps Tendon with Double Femoral and Single Tibial Tunnels: Clinical Results in Two Years Follow Up. Revista Brasileira de Ortopedia (English Edition).

[18] Eguchi A, Adachi N, Nakamae A, Usman MA, Deie M, Ochi M (2014). Proprioceptive function after isolated single-bundle posterior cruciate ligament reconstruction with remnant preservation for chronic posterior cruciate ligament injuries. Orthop Traumatol Surg Res.

[19] Fanelli GC, Beck JD, Edson CJ (2010). Current concepts review: the posterior cruciate ligament. J Knee Surg.

[20] Garofalo R, Jolles BM, Moretti B, Siegrist O (2006). Double-bundle transtibial posterior cruciate ligament reconstruction with a tendon-patellar bone-semitendinosus tendon autograft: clinical results with a minimum of 2 years' follow-up. Arthroscopy.

[21] Gill TJ, DeFrate LE, Wang C (2004). The effect of posterior cruciate ligament reconstruction on patellofemoral contact pressures in the knee joint under simulated muscle loads. Am J Sports Med.

[22] Gill TJ, Van de Velde SK, Wing DW, Oh LS, Hosseini A, Li G (2009). Tibiofemoral and patellofemoral kinematics after reconstruction of an isolated posterior cruciate ligament injury: in vivo analysis during lunge. Am J Sports Med.

[23] Harner CD, Janaushek MA, Ma CB, Kanamori A, Vogrin TM, Woo SL (2000). The effect of knee flexion angle and application of an anterior tibial load at the time of graft fixation on the biomechanics of a posterior cruciate ligament-reconstructed knee. Am J Sports Med.

[24] Hermans S, Corten K, Bellemans J (2009). Long-term results of isolated anterolateral bundle reconstructions of the posterior cruciate ligament: a 6- to 12-year follow-up study. Am J Sports Med.

[25] Ihle C, Ateschrang A, Albrecht D, Mueller J, Stockle U, Schroter S (2014). Occupational consequences after isolated reconstruction of the insufficient posterior cruciate ligament. BMC Res Notes.

[26] Jung YB, Tae SK, Jung HJ, Lee KH (2004). Replacement of the torn posterior cruciate ligament with a mid-third patellar tendon graft with use of a modified tibial inlay method. J Bone Joint Surg Am.

[27] Kennedy NI, LaPrade RF, Goldsmith MT (2014). Posterior cruciate ligament graft fixation angles, part 1: biomechanical evaluation for anatomic single-bundle reconstruction. Am J Sports Med.

[28] Kennedy NI, LaPrade RF, Goldsmith MT (2014). Posterior cruciate ligament graft fixation angles, part 2: biomechanical evaluation for anatomic double-bundle reconstruction. Am J Sports Med.

[29] Kennedy NI, Wijdicks CA, Goldsmith MT (2013). Kinematic analysis of the posterior cruciate ligament, part 1: the individual and collective function of the anterolateral and posteromedial bundles. Am J Sports Med.

[30] Kim YM, Lee CA, Matava MJ (2011). Clinical results of arthroscopic single-bundle transtibial posterior cruciate ligament reconstruction: a systematic review. Am J Sports Med.

[31] Lahner M, Vogel T, von Engelhardt LV, Schulz MS, Strobel MJ (2012). Isolated AL bundle reconstruction of the PCL. Arch Orthop Trauma Surg.

[32] LaPrade CM, Civitarese DM, Rasmussen MT, LaPrade RF (2015). Emerging Updates on the Posterior Cruciate Ligament: A Review of the Current Literature. Am J Sports Med.

[33] Lee DC, Shon OJ, Kwack BH, Lee SJ (2013). Proprioception and clinical results of anterolateral single-bundle posterior cruciate ligament reconstruction with remnant preservation. Knee Surg Relat Res.

[34] Lee YS, Wang JH, Bae JH (2009). Biomechanical evaluation of cross-pin versus interference screw tibial fixation using a soft-tissue graft during transtibial posterior cruciate ligament reconstruction. Arthroscopy.

[35] Li B, Wang JS, He M, Wang GB, Shen P, Bai LH (2015). Comparison of hamstring tendon autograft and tibialis anterior allograft in arthroscopic transtibial single-bundle posterior cruciate ligament reconstruction. Knee Surg Sports Traumatol Arthrosc.

[36] Li G, Gill TJ, DeFrate LE, Zayontz S, Glatt V, Zarins B (2002). Biomechanical consequences of PCL deficiency in the knee under simulated muscle loads–an in vitro experimental study. J Orthop Res.

[37] Li Y, Li J, Wang J, Gao S, Zhang Y (2014). Comparison of single-bundle and double-bundle isolated posterior cruciate ligament reconstruction with allograft: a prospective, randomized study. Arthroscopy.

[38] Lien OA, Aas EJ, Johansen S, Ludvigsen TC, Figved W, Engebretsen L (2010). Clinical outcome after reconstruction for isolated posterior cruciate ligament injury. Knee Surg Sports Traumatol Arthrosc.

[39] Lim HC, Bae JH, Wang JH (2010). Double-bundle PCL reconstruction using tibial double cross-pin fixation. Knee Surg Sports Traumatol Arthrosc.

[40] Lin YC, Chen SK, Liu TH, Cheng YM, Chou pp.  (2013). Arthroscopic transtibial single-bundle posterior cruciate ligament reconstruction using patellar tendon graft compared with hamstring tendon graft. Arch Orthop Trauma Surg.

[41] Liu F, Zhang S, Xiao Y (2022). Stenotic intercondylar notch is not a risk factor for posterior cruciate ligament rupture: a morphological analyses using magnetic resonance imaging. Knee Surg Sports Traumatol Arthrosc.

[42] MacGillivray JD, Stein BE, Park M, Allen AA, Wickiewicz TL, Warren RF (2006). Comparison of tibial inlay versus transtibial techniques for isolated posterior cruciate ligament reconstruction: minimum 2-year follow-up. Arthroscopy.

[43] Mair SD, Schlegel TF, Gill TJ, Hawkins RJ, Steadman JR (2004). Incidence and location of bone bruises after acute posterior cruciate ligament injury. Am J Sports Med.

[44] Mariani PP, Adriani E, Santori N, Maresca G (1997). Arthroscopic posterior cruciate ligament reconstruction with bone-tendon-bone patellar graft. Knee Surg Sports Traumatol Arthrosc.

[45] McAllister DR, Markolf KL, Oakes DA, Young CR, McWilliams J (2002). A biomechanical comparison of tibial inlay and tibial tunnel posterior cruciate ligament reconstruction techniques: graft pretension and knee laxity. Am J Sports Med.

[46] Mygind-Klavsen B, Nielsen TG, Lind MC (2017). Outcomes After Posterior Cruciate Ligament (PCL) Reconstruction in Patients With Isolated and Combined PCL Tears. Orthop J Sports Med.

[47] Noh JH, Yoon KH, Kyung HS, Roh YH, Kang TS (2017). Multiple looping technique for tibial fixation in posterior cruciate ligament reconstruction using free tendon Achilles allograft. Knee Surg Sports Traumatol Arthrosc.

[48] Norbakhsh ST, Zafarani Z, Najafi A, Aslani H (2014). Arthroscopic posterior cruciate ligament reconstruction by using hamstring tendon autograft and transosseous screw fixation: minimal 3 years follow-up. Arch Orthop Trauma Surg.

[49] Ochiai S, Hagino T, Senga S, Yamashita T, Haro H (2019). Treatment Outcome of Reconstruction for Isolated Posterior Cruciate Injury: Subjective and Objective Evaluations. J Knee Surg.

[50] Owesen C, Aas E, Aroen A (2018). Surgical reconstruction is a cost-efficient treatment option for isolated PCL injuries. Knee Surg Sports Traumatol Arthrosc.

[51] Pache S, Aman ZS, Kennedy M (2018). Posterior Cruciate Ligament: Current Concepts Review. Arch Bone Jt Surg.

[52] Page MJ, McKenzie JE, Bossuyt PM (2021). The PRISMA 2020 statement: an updated guideline for reporting systematic reviews. BMJ.

[53] Panchal HB, Sekiya JK (2011). Open tibial inlay versus arthroscopic transtibial posterior cruciate ligament reconstructions. Arthroscopy.

[54] Papannagari R, DeFrate LE, Nha KW (2007). Function of posterior cruciate ligament bundles during in vivo knee flexion. Am J Sports Med.

[55] Rauck RC, Nwachukwu BU, Allen AA, Warren RF, Altchek DW, Williams RJ (2019). Outcome of isolated posterior cruciate ligament reconstruction at mean 6.3-year follow up: a consecutive case series. Phys Sportsmed.

[56] Rhatomy S, Abadi MBT, Setyawan R (2021). Posterior cruciate ligament reconstruction with peroneus longus tendon versus hamstring tendon: a comparison of functional outcome and donor site morbidity. Knee Surg Sports Traumatol Arthrosc.

[57] Sanders TL, Pareek A, Barrett IJ (2017). Incidence and long-term follow-up of isolated posterior cruciate ligament tears. Knee Surg Sports Traumatol Arthrosc.

[58] Saragaglia D, Francony F, Gaillot J, Pailhe R, Rubens-Duval B, Lateur G (2020). Posterior cruciate ligament reconstruction for chronic lesions: clinical experience with hamstring versus ligament advanced reinforcement system as graft. Int Orthop.

[59] Schroven W, Vles G, Verhaegen J, Roussot M, Bellemans J, Konan S (2022). Operative management of isolated posterior cruciate ligament injuries improves stability and reduces the incidence of secondary osteoarthritis: a systematic review. Knee Surg Sports Traumatol Arthrosc.

[60] Schulz MS, Russe K, Weiler A, Eichhorn HJ, Strobel MJ (2003). Epidemiology of posterior cruciate ligament injuries. Arch Orthop Trauma Surg.

[61] Schumaier A, Minoughan C, Jimenez A, Grawe B (2019). Treatments of Choice for Isolated, Full-Thickness Tears of the Posterior Cruciate Ligament: A Nationwide Survey of Orthopaedic Surgeons. J Knee Surg.

[62] Sekiya JK, West RV, Ong BC, Irrgang JJ, Fu FH, Harner CD (2005). Clinical outcomes after isolated arthroscopic single-bundle posterior cruciate ligament reconstruction. Arthroscopy.

[63] Seon JK, Song EK (2006). Reconstruction of isolated posterior cruciate ligament injuries: a clinical comparison of the transtibial and tibial inlay techniques. Arthroscopy.

[64] Shin YS, Kim HJ, Lee DH (2017). No Clinically Important Difference in Knee Scores or Instability Between Transtibial and Inlay Techniques for PCL Reconstruction: A Systematic Review. Clin Orthop Relat Res.

[65] Shon OJ, Lee DC, Park CH, Kim WH, Jung KA (2010). A comparison of arthroscopically assisted single and double bundle tibial inlay reconstruction for isolated posterior cruciate ligament injury. Clin Orthop Surg.

[66] Song EK, Park HW, Ahn YS, Seon JK (2014). Transtibial versus tibial inlay techniques for posterior cruciate ligament reconstruction: long-term follow-up study. Am J Sports Med.

[67] Sun X, Zhang J, Qu X, Zheng Y (2015). Arthroscopic posterior cruciate ligament reconstruction with allograft versus autograft. Arch Med Sci.

[68] Swenson DM, Collins CL, Best TM, Flanigan DC, Fields SK, Comstock RD (2013). Epidemiology of knee injuries among US high school athletes, 2005/2006–2010/2011. Med Sci Sports Exerc.

[69] Tachibana Y, Tanaka Y, Kazutaka K, Horibe S (2021). Second-look arthroscopy after double-bundle posterior cruciate ligament reconstruction: Effect of patient age. Asia Pac J Sports Med Arthrosc Rehabil Technol.

[70] Tian P, Hu WQ, Li ZJ, Sun XL, Ma XL (2017). Comparison of autograft and allograft tendons in posterior cruciate ligament reconstruction: a meta-analysis. Medicine (Baltimore).

[71] van Kuijk KSR, Reijman M, Bierma-Zeinstra SMA, Waarsing JH, Meuffels DE (2019). Posterior cruciate ligament injury is influenced by intercondylar shape and size of tibial eminence. Bone Joint J.

[72] Wang CJ, Chan YS, Weng LH, Yuan LJ, Chen HS (2004). Comparison of autogenous and allogenous posterior cruciate ligament reconstructions of the knee. Injury.

[73] Wijdicks CA, Kennedy NI, Goldsmith MT (2013). Kinematic analysis of the posterior cruciate ligament, part 2: a comparison of anatomic single- versus double-bundle reconstruction. Am J Sports Med.

[74] Wind WM, Bergfeld JA, Parker RD (2004). Evaluation and treatment of posterior cruciate ligament injuries: revisited. Am J Sports Med.

[75] Winkler PW, Zsidai B, Wagala NN (2021). Evolving evidence in the treatment of primary and recurrent posterior cruciate ligament injuries, part 2: surgical techniques, outcomes and rehabilitation. Knee Surg Sports Traumatol Arthrosc.

[76] Wong T, Wang CJ, Weng LH (2009). Functional outcomes of arthroscopic posterior cruciate ligament reconstruction: comparison of anteromedial and anterolateral trans-tibia approach. Arch Orthop Trauma Surg.

[77] Wu CH, Chen AC, Yuan LJ (2007). Arthroscopic reconstruction of the posterior cruciate ligament by using a quadriceps tendon autograft: a minimum 5-year follow-up. Arthroscopy.

[78] Xu X, Huang T, Liu Z (2014). Hamstring tendon autograft versus LARS artificial ligament for arthroscopic posterior cruciate ligament reconstruction in a long-term follow-up. Arch Orthop Trauma Surg.

[79] Yang JH, Yoon JR, Jeong HI (2012). Second-look arthroscopic assessment of arthroscopic single-bundle posterior cruciate ligament reconstruction: comparison of mixed graft versus achilles tendon allograft. Am J Sports Med.

[80] Yoon KH, Kim EJ, Kwon YB, Kim SG (2019). Minimum 10-Year Results of Single- Versus Double-Bundle Posterior Cruciate Ligament Reconstruction: Clinical, Radiologic, and Survivorship Outcomes. Am J Sports Med.

[81] Zawodny SR, Miller MD (2010). Complications of posterior cruciate ligament surgery. Sports Med Arthrosc Rev.

[82] Zayni R, Hager JP, Archbold P (2011). Activity level recovery after arthroscopic PCL reconstruction: a series of 21 patients with a mean follow-up of 29 months. Knee.

[83] Zhao J, Huangfu X (2007). Arthroscopic single-bundle posterior cruciate ligament reconstruction: Retrospective review of 4- versus 7-strand hamstring tendon graft. Knee.

[84] Zhao J, Xiaoqiao H, He Y, Yang X, Liu C, Lu Z (2008). Sandwich-style posterior cruciate ligament reconstruction. Arthroscopy.

[85] Zhao JZ, Huang-Fu XQ, He YH, Yang XG (2009). Single-bundle posterior cruciate ligament reconstruction with remnant preservation: lateral versus medial-sided augmentation technique. Orthop Surg.

