# Supplementary material for: Global variation in isolated posterior cruciate ligament reconstruction
Source: J Exp Orthop. 2022 Oct 9;9:104. doi: 10.1186/s40634-022-00541-4 (PMC9548455; doi:10.1186/s40634-022-00541-4)
Supplement: Supplementary file 1 — Additional file 1: Table S1. Overview of included studies based on global region. [file 40634_2022_541_MOESM1_ESM.docx]

Additional file 1: Table S1. Overview of Included Studies Based on Global Region

**Asia**

| **Author (Y)** | **Level of Evidence** | **Region (Country)** | **# Patients**  **[Sex]** | **Mean Age (range) (Y)** | **Injury Mechanism** | **Single v. Double Bundle** | **Graft Type**  **(Size) (cm)** | **Graft Source** | **Tibial Technique** | **Tibial Fixation** | **Femoral Fixation** | **Femoral Tunnel Drilling** | **PCL Remnant** |
| --- | --- | --- | --- | --- | --- | --- | --- | --- | --- | --- | --- | --- | --- |
| Adachi et al. ^1^ (2007) | 3 | Asia (Japan) | 29  [22M, 7F] | 31.9 (17-54) | 14 MVA, 13 sports, 2 falls | Single | 3/4-strand HT (75mm) | Auto | TT | Double spike staples | Endobutton | Inside-Out | NR |
| Ahn et al. ^4^ (2006) | 4 | Asia (Korea) | 61  [45M, 16F] | 30.4 (20-51) | 32 MVA, 17 sports, 10 direct blow, 2 falls | Single | 2-loop HT (n=21)  AT (n=40) | Auto (n=21)  Allo  (n=40) | TT | Bioabsorbable screw | Bioabsorbable interference screw | NR | PM bundle preserved |
| Ahn et al. ^3^ (2013) | 4 | Asia (Korea) | 30  [27M, 3F] | 36 (21-56) |  | Single | TA (n=22)  4-strand HT (n=8) | Auto (n=8)  Allo (n=22) | TT | Screw and washer | Bio-interference screw | Outside-In | Preserved |
| Chan et al. ^12^ (2006) | 4 | Asia (Taiwan) | 20  [15M, 5F] | 29 (20-57) | 17 MVA, 3 sports | Single | 4-strand HS (24) | Auto | TT | Biocortical screw + washer | BioScrew (Linvatec) | Outside-In | Small remnant left |
| Chen et al. ^14^ (2002) | 4 | Asia (Taiwan) | 54  [32M, 14F] | QT Group: 29  HT Group: 27 |  | Single | QT (8) (n=22)  HT (10) (n=27) | Auto | TT | Biocortical screw + washer | Cancellous screw and washer | Outside-In | NR |
| Chen et al. ^13^ (2009) | 4 | Asia (China) | 22  [17M, 5F] | 39 (19-53) |  | Double | 4-strand HT | Auto | TT | Braiding sutures with transverse tunnel | No.5 polyester sutures tied over bone bridge | Inside-Out | Femoral insertion side preserved |
| Chen et al. ^15^ (2012) | 4 | Asia (Taiwan) | 38  [23M, 15F] | 32.6 (19-59) | 32 MVA, 6 sports | Double | LARS | Artificial | TT | Metal interference screw | Metal interference screw | Outside-In | Preserved |
| Eguchi et al. ^18^ (2014) | 4 | Asia (Japan) | 19  [16M, 3F] | 27.9 (17-59) | 12 MVA, 6 sports, 1 fall | Single | HT (75mm) | Auto | TT | Double spike staples | EndoButton | Inside-Out | Preserved |
| Jung et al. ^26^ (2004) | 4 | Asia (Korea) | 12  [11M, 1F] | 29 (17-56) | 5 MVA, 4 sports, 3 falls | Single | BPTB | Auto | TI | Hybrid fixation with suture backup | Biodegradable interference fit | NR | Preserved |
| Lee et al. ^33^ (2013) | 3 | Asia (Korea) | 20  [16M, 4F] | 36 (17-60) | 12 MVA, 4 sports, 4 direct impact | Single | AT | Auto | TT | Bioabsorbable screw + cancellous screw and washer | Absorbable interference screw | Inside-Out | Preserved |
| Li et al. ^37^ (2014) | 3 | Asia (China) | 46  [33M, 13F] | SB Group: 25.1  DB Group: 23.5 | 18 sports, 22 MVA, 6 falls | Single (n=22)  Double(n=24) | TA | Allo | TT | Bioabsorbable screw | EndoButton | NR | Preserved |
| Li et al.^35^  (2015) | 3 | Asia (China) | 37  [25M, 13F] | 31.8 (18-55) | 14 MVA, 12 sports, 9 daily activities, 2 work-related | Single | 4-strand HT (11.4) (n=18)  2-strand TA (14.4) (n=19) | Auto (n=18)  Allo (n=19) | TT | Cannulated Interference screw | EndoButton | Inside-Out | Stump Retained |
| Lim et al. ^40^ (2010) | 4 | Asia (Korea) | 22  [19M, 3F] | 36 (18-59) | 12 sports, 8 MVA, 2 falls | Double | AT (10) | Auto | TT | Two bioabsorbable cross-pins | Bioabsorbable interference screw | Inside-Out | Debrided |
| Lin et al.^41^ (2013) | 3 | Asia (Taiwan) | 59  [44M, 15F] | PT Group: 26.8 (15-52)  HT Group: 26.2 (16-44) | 50 MVA, 9 sports | Single | PT (n=25)  HT (n=34) | Auto | TT | Metal interference screw (n=25)  Bioabsorbable screw (n=34) | Interference screw | Outside-In | Preserved |
| Noh et al. ^48^ (2017) | 4 | Asia (Korea) | 32  [29M, 3F] | 30.5 (19-48) | 10 falls, 10 MVA, 9 sports, 3 unknown | Single | 2-strand AT (11-11.5) | Allo | TT | Multiple looping technique | Suture washer with 4 pairs of tethering sutures | Inside-Out | Preserved |
| Norbakhsh et al.^49^ (2014) | 4 | Asia (Iran) | 52  [42M, 10F] | 27 (18-49) |  | Single | 4-strand HT | Auto | TT | Biointerference screw | Transcrew fixation | NR | NR |
| Ochiai et al. ^50^ (2018) | 3 | Asia (Japan) | 23  [18M, 5F] | 28.9 (16-47) | 11 MVA, 9 sports, 3 falls | Single | 4-strand ST (min. 65mm) | Auto | TT | Double spike staples | Endobutton | Outside-In | NR |
| Rhatomy et al. ^56^ (2021) | 2 | Asia (Indonesia) | 55  [33M, 22F] | 29.69 (18-45) | 13 MVA, 27 sports, 15 other | Single | HT (8.3)(n=27)  PLT (8.2)(n=28) | Auto | TT | Bio-absorbable screw | Button (Graftmax) | Inside-Out | Preserved |
| Seon et al. ^63^ (2006) | 3 | Asia (Korea) | 43  [36M, 7F] | Group A: 29.1 (17-56)  Group B: 29.4 (18-50) | 29 sports, 13 MVA, 1 fall | Single | HT (n=21)  BPTB (n=22) | Auto | TT (n=21)  TI (n=22) | Interference Screw (TT group)  Screw + washer/staple (TI group) | LA screw (TT group)  Interference Screw (TI group) | Inside-Out | NR |
| Shon et al. ^65^ (2010) | 4 | Asia (Korea) | 30 [26M, 4F] | Group A: 34 (20-61)  Group B: 36 (18-52) | 23 MVA, 5 sports, 2 falls | Single (n=14)  Double (n=16) | BPTB (n=10)  AT (n=20) | Allo | TI | Cancellous screw + washer | Interference screw + staple | Outside-In | NR |
| Song et al. ^66^ (2014) | 3 | Asia (Korea) | 66  [58M, 8F] | TT Group: 37  TI Group: 35 | 41 sports, 20 MVA, 5 falls | Single | BPTB (n=30)  HT (n=36) | Auto | TT (n=36)  TI (n=30) | Biointerference screw (TT group)  Screw and washer (TI group) | LA screw (TT group)  Interference screw (TI group) | Inside-Out | NR |
| Tachibana et al. ^69^ (2021) | 3 | Asia (Japan) | 26  [24M, 2F] | 36 (17-49) | 12 sports, 9 MVA, 5 work-related | Double | Tripled HT (8-85 mm) | Auto | TT | Double-spike plates (x2) | Endobutton (x2) | Inside-Out | Cleared |
| Wang et al. ^72^ (2004) | 2 | Asia (Taiwan) | 55  [41M, 14F] | Autograft group: 29 (16-54)  Allograft group: 30 (16-64) | 51 MVA, 4 sports | Single | QT (n=16)  HT (n=16)  AT (n=14)  TA (n=9) | Auto (n=32)  Allo  (n=23) | TT | Titanium screw for QT and AT  Bioabsorbable screw for HS and TA | Bio-absorbable screw | Outside-In | NR |
| Wong et al. ^76^ (2009) | 3 | Asia (Taiwan) | 55  [41M, 14F] | 30 (16-60) | 51 MVA, 4 sports | Single | ST | Auto | TT | Bioabsorbable screw | Biodegradable screw | Inside-Out | Retained |
| Wu et al. ^77^ (2007) | 4 | Asia (China) | 22  [17M, 5F] | 27 (18-49) | 17 MVA, 3 sports, 2 falls | Single | QT (25mm) | Auto | TT | Biocortical screw & washer | Titanium interference screw | Outside-In | NR |
| Xu et al. ^78^ (2014) | 3 | Asia (China) | 35  [17M, 18F] | HS Group: 29.1  LARS Group: 28.6 |  | Single | HT (12)(n=16)  LARS (n=19) | Auto (n=16)  Artificial (n=19) | TT | Screw | Titanium interference screw | Outside-In | HS Group: Portion left  LARS Group: Resected |
| Yang et al. ^79^ (2012) | 2 | Asia (Korea) | 58  [49M, 9F] | Group A: 27.7  Group B: 26 | 17 MVA, 18 sports, 18 daily-activities, 4 work-related | Single | TA + HT (17-18)(n=30)  AT (14-15)(n=28) | Auto (n=30)  Allo (n=58)  Auto (n=30) | TT | Interference screw + post tie reinforcement | Interference screw, post tie reinforcement (Group A) | Outside-In | Retained |
| Yoon et al. ^80^ (2019) | 3 | Asia (Korea) | 64  [55M, 9F] | SB Group: 29.1 (15-58)  DB Group: 27.0 (18-48) |  | Single (n=28)  Double (n=36) | AT | Allo | TT | Interference screw | Cancellous screw + spiked washer/staples, interference screw (double fixation) | NR | Preserved |
| Zhao et al. ^83^ (2007) | 3 | Asia (China) | 51  [34M, 9F] | 4SHG Group: 23-46  7SHG Group: 19-45 |  | Single | 4-strand HT (n=25)  7-strand HT (n=26) | Auto | TT | Titanium button or screw post | Mini-plate | NR | Debrided |
| Zhao et al. ^84^ (2008) | 4 | Asia (China) | 18  [14M, 4F] | 34 (19-42) |  | Double | 8-strand HT | Auto | TT | Titanium button | Mini-plate | Inside-Out | Preserved |
| Zhao et al. ^85^ (2009) | 2 | Asia (China) | 42  [33M, 9F] | MSA Group: 29 (24-47)  LSA Group: 28 (22-49) |  | Single | 7-strand HT (7) | Auto | TT | Titanium Button | Mini-plate | Inside-Out | Preserved |

**Europe**

| Boutefnouchet  et al. ^9^ (2013) | 3 | Europe  (United Kingdom) | 15  [15M, 0F] | 25 (19-38) | 12 sports, 3 MVA | Single | 4-strand HT  (24) | Auto | TT | Barbed staples x 2 | Cancellous screw | Inside-Out | Resected |
| --- | --- | --- | --- | --- | --- | --- | --- | --- | --- | --- | --- | --- | --- |
| Garofalo et al. ^20^ (2006) | 4 | Europe (Italy) | 15  [14M, 1F] | 27.2 (17-43) | 11 MVA, 4 sports | Double | BPTB, ST (10) | Auto | TT | Bioresorbable interference screw | Bioresorbable interference screw | Outside-In | Debrided |
| Hermans et al. ^24^ (2009) | 4 | Europe (Belgium) | 25  [22M, 3F] | 30.8 (17-52) | 13 sports, 7 MVA, 5 work-related | Single | BPTB (n=9)  4-strand HT (n=7)  2-strand HT + AT (n=8)  AT (n=1) | Auto | TT | Interference screw, back-up staple fixation | Cannulated interference screw | Outside-In | NR |
| Ihle et al. ^25^ (2014) | 4 | Europe (Germany) | 12  [10M, 2F] | 28.2 (15-43) | 8 sports, 3 MVA, | Single | ST | Auto | TT | Interference screw | Endobutton | NR | NR |
| Lahner et al. ^31^ (2012) | 4 | Europe (Germany) | 33  [17M, 16F] | 32.5 (18.3-53.7) | 17 MVA, 12 sports, 1 work, 1 unknown | Single | HT (10-11) | Auto | TT | Biodegradable Interference screw + fixation button | Biodegradable interference screw | Inside-Out | Preserved |
| Lien et al. ^39^ (2010) | 3 | Europe (Norway) | 43  [29M, 14F] | 28(17-53) |  | Single (n=17)  Double (n=7) | BPTB (n=19)  HT (n=24) | Auto | TT | NR | NR | Inside-Out | NR |
| Mariani et al. ^45^ (1997) | 4 | Europe (Italy) | 24  [16M, 8F] | 26(16-39) |  | Single | BPTB | Auto | TT | 7 or 9 mm screw | Cannulated interference screw | Outside-In | Removed |
| Saragaglia et al. ^58^ (2020) | 3 | Europe (France) | 16  [15M, 1F] | 29.3 (17-54) | 6 sports, 11 MVA | Double | HT (16-18) (n=8)  LARS (n=8) | Auto (n=8)  Artificial (n=8) | TT | Interference screw | Interference screw + 2 serrated staples | Outside-In | NR |
| Zayni et al. ^82^ (2011) | 4 | Europe (France) | 21  [18M, 3F] | 29 (18-46) | 15 sports, 6 MVA | Single | QT (10-12) | Auto | TT | Resorbable interference screw | Resorbable interference screw + traction wire tied around bicortical screw | Outside-In | Resected |

**North America**

| Gill et al. ^22^ (2009) | 4 | N. America (USA) | 7  [5M, 2F] | 35 (20-51) |  | Single | AT (10mm) | Allo | TT | Bioscrew & titanium interference screw | Titanium interference screw | Outside-In | Debrided |
| --- | --- | --- | --- | --- | --- | --- | --- | --- | --- | --- | --- | --- | --- |
| MacGillivray et al. ^43^ (2006) | 3 | N. America (USA) | 20  [15M, 5F] | Group 1: 29 (17-49(  Group 2: 31 (23-39) | 11 sports, 6 MVA, 3 falls | Single | BPTB (n=18)  AT (n=2) | Auto (n=11)  Allo (n=9) | TT (n=13)  TI (n=7) | NR | Interference screw | Outside-In | NR |
| Rauck et al. ^55^ (2019) | 4 | N. America (USA) | 15  [8M, 6F] | 27.5 (17-43) | 4 MVA, 8 sports, 1 fall, 1 unknown | Single (n=12)  Double (n=3) | QT (n=7)  AT (n=6)  HT(n=2) | Auto  (n=9)  Allo (n=6) | TT (n=12)  TI (n=3) | NR | NR | NR | NR |
| Sekiya et al. ^62^ (2005) | 4 | N. America (USA) | 21  [15M, 6F] | 38 (20-62) |  | Single | AT | Auto | TT | Screw and soft tissue washer | Interference Screw | Outside-In | Debrided |

**South America**

| Cury et al. ^17^ (2012) | 4 | S. America (Brazil) | 14  [9M, 5F] | 31 (26-43) | 9 MVA, 5 sports | Double | ST (PM), QT (AL) | Auto | TT | Cortical screw | Interference screw | Outside-In | Removed |
| --- | --- | --- | --- | --- | --- | --- | --- | --- | --- | --- | --- | --- | --- |

Legend: y, years; m, months; cm, centimeters; mm, millimeters; NR, not recorded; HS, hamstring tendon; ST, semitendinosus; AT, Achilles tendon; QT, quadriceps tendon; TA, tibialis anterior; PLT, peroneus longus tendon; PT, patellar tendon; LARS, ligament augmentation and reconstruction system; BPTB, bone-patellar-tendon-bone; PM, posteromedial bundle; AL, anterolateral bundle; TT- trans-tibial; TI; tibial inlay; MVA, motor vehicle accident; Auto, autograft tendon; allo, allograft tendon; N, North; S, South; M, male patient; F, female patient; 4SHG, 4-strand hamstring graft; 7SHG, 7-strand hamstring graft; MSA, medial-sided augmentation; LSA, lateral-sided augmentation.
