# Supplementary material for: Global variation in isolated posterior cruciate ligament reconstruction
Source: J Exp Orthop. 2022 Oct 9;9:104. doi: 10.1186/s40634-022-00541-4 (PMC9548455; doi:10.1186/s40634-022-00541-4)
Supplement: Supplementary file 2 — Additional file 2: Table S2. Outcomes following isolated PCL reconstruction. [file 40634_2022_541_MOESM2_ESM.docx]

Additional file 2: Table S2. Outcomes Following Isolated PCL Reconstruction

| Author (Y) | Mean Follow Up (M) | Patient Reported Outcomes | Complications |
| --- | --- | --- | --- |
| Adachi et al. ^1^ (2007) | 42 | Posterior laxity (arthrometer)  Pre-Op: 9.3 +- 2.2 mm  Post-Op: 3.7 +- 2.4 mm | NR |
| Ahn et al. ^4^ (2006) | 40.8 (24-84) | Lysholm  Pre-Op: 65.8 +- 10.8  Post-Op: 92.9 +- 3.5  IKDC  Pre-Op: 32-C, 29-D  Post-Op: 33-A, 26-B  Side to side difference (Arthrometer)  Pre-Op: 12.95 +- 3.14 mm  Post-Op: 2.79 +- 1.44 mm | NR |
| Ahn et al. ^3^ (2013) | 47 (25-62) | Lysholm  Pre-Op: 61.5 +- 13.5  Post-Op: 88 +- 4.3  IKDC  Pre-Op: 63.3 +- 11.6; 30-D  Post-Op: 87 +- 4.6; 11-A, 17-B, 2-C  Side to side differences (Telos)  Pre-Op: 13.4 +- 3.1 mm  Post-Op: 3.2 +- 1.5 mm | Arthrofibrosis (n=1)  Mild infection (n=2)  Tibial tunnel complication (n=7) |
| Boutefnouchet  et al. ^9^ (2013) | 12 | Post-Op:  Lysholm: 90.67 +- 21.37  Tegner: 6.33 +_ 2.02  IKDC: 10-A, 4-B, 1-C | Serous drainage (n=2)  Complex regional pain syndrome (n=1) |
| Chan et al. ^12^ (2006) | 40 | Lysholm  Pre-Op: 63 +- 10  Post-Op: 93 +- 9  Tegner  Pre-Injury: 7 +- 1.5  Pre-Op: 3 +- 1.9  Post-Op: 6.3 +- 2.4  IKDC  Pre-Op: 6-C, 14-D  Post-Op: 5-A, 12-B, 2-C, 1-D  Posterior Drawer  Pre-Op: 15 Grade 3, 5 Grade 4  Post-Op: 16 Grade 1, 3 Grade 2, 1 Grade 3  Posterior Displacement (KT-1000)  Pre-Op: 12 +- 3.4 mm  Post-Op: 3.8 +- 2.5 mm | Stitch abscess at tibial site (n=1)  Screw head protrusion (n=1) |
| Chen et al. ^14^ (2002) | QT Group: 30 (24-36)  HT Group: 26 (24-30) | Lysholm  QT Group Post-Op: 90.63 +- 7.74  HT Group Post-Op: 91.44 +- 6.17  IKDC  QT Group Post-Op: 5-A, 13-B, 3-C, 1-D  HT Group Post-Op: 7-A, 15-B, 4-C, 1-D  Arthrometer Post-Op  QT Group: 3.72 +- 1.66 mm  HT Group: 4.11 +- 1.60 mm | Superficial wound infection (n=2)  Hardware removal (n=8) |
| Chen et al. ^13^ (2009) | 24 months min. | Lysholm  Pre-Op: 63.5 +- 4.9  Post-Op: 92.5 +- 4.1  Tegner  Pre-Op: 5.1  Post-Op: 6.3  IKDC  Pre-Op: 65.6 +- 5.1; All abnormal/severely abnormal  Post-Op: 92.1 +- 3.7; 15-A, 3-B, 1-C  Arthrometer  Pre-Op: 10.6 +- 2.0 mm  Post-Op: 2.0 +- 1.2 mm  Posterior Drawer  Pre-Op: 15 Grade 2, 4 Grade 3  Post-Op: 17 negative, 1 Grade 1, 1 Grade 2 | None |
| Chen et al. ^15^ (2012) | 37 (30-68) | Lysholm  Pre-Op: 70.0 +- 11.0  Post-Op: 91.7 +- 5.5  Tegner  Pre-Op: 3.4 +- 0.6  Post-Op: 6.0 +- 1.4  IKDC  Pre-Op: 36-C, 2-D  Post-Op: 7-A, 27-B, 2-C, 2-D  Arthrometer  Pre-Op: 11.5 +- 0.5 mm  Post-Op: 4.2 +- 1.1 mm | Synovitis (n=1)  Superficial wound infection (n=1)  Deep infection (n=1) |
| Cury et al. ^17^ (2012) | 24 | Lysholm  Pre-Op: 66  Post-Op: 93  IKDC  Pre-Op: 4-C, 10-D  Post-Op: 3-A, 10-B, 1-C  Posterior Drawer  Pre-Op: 5 Grade 2, 9 Grade 3  Post-Op: 8 Normal, 5 Grade 1, 1 Grade 2  Side-to-side difference (KT-1000)  Pre-Op: 5 6-10 mm, 9 > 10mm  Post-Op: 8 0-2 mm, 5 3-5 mm, 1 6-10 mm | Pain (n=2) |
| Eguchi et al. ^18^ (2014) | 27.1 (24-57) | Lysholm  Pre-Op: 63.7 +- 13.2  Post-Op: 94.4 +- 4.6  Posterior laxity (KT-2000)  Pre-Op: 6.4 +- 3.4 mm  Post-Op: 1.0 +- 1.8 mm | NR |
| Garofalo et al. ^20^ (2006) | 38.4 (24-60) | Lysholm  Pre-Op: 61  Post-Op: 87.5  Tenger  Pre-Injury: 7.9  Pre-Op: 2.1  Post-Op: 6.2  IKDC  Pre-Op: 2-C, 13-D  Post-Op: 1-A, 8-B, 4-C, 2-D  Post. Drawer  Pre-Op: 15 Grade 3  Post-Op: 3 Normal, 10 Grade 1, 2 Grade 2  Posterior translation (Telos)  Pre-Op: 15.7 mm  Post-Op: 8.06 mm | Superficial suture abscess (n=2)  Hypersensitive anterior scar & trouble kneeling (n=4) |
| Gill et al. ^22^ (2009) | 24 | IKDC  Pre-Op: 43.1 +- 15.5  Post-Op: 83.5 +- 7.6 | NR |
| Hermans et al. ^24^ (2009) | 109.2 (78-151.2) | Lysholm  Pre-Op: 50 +- 23  Post-Op: 75 +- 20  Tegner  Pre-Injury: 7.2 +- 1.8  Post-Op: 5.7 +- 2  IKDC  Pre-Op: 38 +- 17  Post-Op: 65 +- 24  Post-Op: 9-A, 8-B, 13-C  Post. Translation (Telos)  Post-Op: 8.6 +- 2.9 mm  Post. Drawer  Post-Op: 2 Normal, 15 Grade 1, 5 Grade 2 | Residual soreness requiring hardware removal (n=4)  Open capsular release and decreased ROM (n=1) |
| Ihle et al. ^25^ (2014) | 51 (14-75) | Lysholm  Pre-Op: 46.4 +- 17.3  Post-Op: 84.7 +- 14.1  Tegner  Post-Op: 4.8 +- 1.2  IKDC  Post-Op: 80.0 +- 16.2 | Occasional knee pain (n=6)  Regular use of analgesics (n=2) |
| Jung et al. ^26^ (2004) | 5.4 (1-10) | IKDC  Post-Op: 4-A, 7-B  Side to side difference (Telos)  Pre-Op: 9.0 +- 2.1 mm  Post-Op: 1.8 +- 1.2 mm | Hardware removal (n=4) |
| Lahner et al. ^31^ (2012) | 23.8 (12.2-34.2) | Tegner  Pre-Op: 2.8 +- 0.8  Post-Op: 5.9 +- 1.2  IKDC  Pre-Op: 41.86 +- 11.49; 2-B, 17-C, 14-D  Post-Op: 69.54 +- 11.39; 5-A, 19-B, 8-C, 1-D  Posterior Translation (Telos)  Pre-Op: 10.1 +- 1.8 mm  Post-Op: 5.0 +- 2.5 mm | Mild Post-Op effusion (n=1) |
| Lee et al. ^33^ (2013) | 61.3 (31-92) | Lysholm  Pre-Op: 70.00 +- 6.89  Post-Op: 88.9 +- 4.36  Tegner  Pre-Op: 2.70 +- 0.92  Post-Op: 6.20 +- 1.32  IKDC  Pre-Op: 62.72 +- 10.51; 12-C, 8-D  Post-Op: 85.41 +- 7.91; 8-A, 12-B  Post. Drawer  Pre-Op: 12 Grade 2, 8 Grade 3  Post-Op: 20 Grade 1 | NR |
| Li et al. ^37^ (2014) | SB Group: 28.7  DB Group: 30.4 | Lysholm  SB Group Pre-Op: 63.1 +- 3.8  Post-Op: 88.0 +- 4.2  DB Group Pre-Op: 64.6 +- 4.3  Post-Op: 89.8 +- 3.8  Tegner  SB Group Pre-Op: 3.1 +- 0.6  Post-Op: 6.2 +- 0.9  DB Group Pre-Op: 3.3 +- 1.0  Post-Op: 6.8 +- 1.2  IKDC  SB Group Post-Op: 65.5 +- 7.8; 10-A, 8-B, 3-C, 1-D  DB Group Post-Op: 71.6 +- 6.7; 13-A, 9-B, 2-C.  Side to side difference (arthrometer)  SB Group Pre-Op: 9.6 +- 0.9 mm  Post-Op: 4.1 +- 1.3 mm  DB Group Pre-Op: 9.6 +- 1.5 mm  Post-Op: 2.2 +- 1.3 mm | None |
| Li et al.^35^  (2015) | HG Group: 27.6  TA Group: 28.8 | Lysholm  HG Pre-Op: 65  Post-Op: 84  TA Pre-Op: 66  Post-Op: 85  Tegner  Pre-Op: 2  Post-Op: 6  IKDC  Pre-Op: 21-D, 16-C  Post-Op: 11-A, 17-B, 7-C, 2-D  Knee laxity (arthrometer)  HG Pre-Op: 11.7 +- 1.9 mm  Post-Op: 4.1 +- 1.7 mm  TA Pre-Op: 11.9 +- 1.7 mm  Post-Op: 3.3 +- 1.8 mm  Post. Drawer Test  Pre-Op: 6 Grade 2, 31 Grade 3  Post-Op: 11 Grade 0, 20 Grade 1, 6 Grade 2 | Paresthesia on medial side of knee (n=2)  Uncomfortable on medial knee during activities (n=7) |
| Lien et al. ^39^ (2010) | 48 (17-109) | Lysholm  Post-Op: 80  Tegner  Pre-Injury: 7  Post-Op: 6  IKDC  Post-Op: 63  KT 1000  Post-Op: 9.2 mm | NR |
| Lim et al. ^40^ (2010) | 33 (24-60) | Lysholm  Pre-Op: 64  Post-Op: 88  Tegner  Pre-Injury: 7  Pre-Op: 3  Post-Op: 6  IKDC  Pre-Op: All patients C/D  Post-Op: 20-A/B, 2-C  Side to side difference (arthrometer)  Pre-Op: 11 mm  Post-Op: 3 mm | None |
| Lin et al.^41^ (2013) | PT Group: 51.6 (36-74)  HT Group: 51.1 (36-67) | Lysholm  PT Group Pre-Op: 63.1 +- 8.8  Post-Op: 91.9 +- 4.3  HT Group Pre-Op: 59.9 +- 10.9  Post-Op: 93.1 +- 3.9  IKDC  PT Group Pre-Op: 3-C, 22-D  Post-Op: 4-A, 17-B, 4-C  HT Group Pre-Op: 3-C, 31-D  Post-Op: 12-A, 20-B, 2-C  Side to side difference (arthrometer)  PT Group Pre-Op: 11.2 +- 1.8 mm  Post-Op: 2.8 +- 1.6 mm  HT Group Pre-Op: 11.1 +- 1.6 mm  Post-Op: 2.6 +- 1.5 mm  Posterior Drawer  PT Group Pre-Op: 3 Grade 2, 22 Grade 3  Post-Op: 4 Grade 0, 17 Grade 1, 4 Grade 2  HT Group Pre-Op: 3 Grade 2, 31 Grade 3  Post-Op: 16 Grade 0, 16 Grade 1, 2 Grade 2. | None |
| MacGillivray et al. ^43^ (2006) | Group 1: 6.3y (2.4-15)  Group 2: 4.7y (2-7) | Lysholm  Group 1 Post-Op: 81.45 +- 13.6  Group 2 Post-Op: 75.7 +-19.5  Tegner  Group 1 Pre-Op: 6.92  Group 2 Pre-Op: 6.86  Post-Op: 6  Posterior Drawer  Group 1 Pre-Op: 5-B, 8-C  Post-Op: 3-A, 6-B, 4-C  Group 2 Pre-Op: 3-B, 4-C  Post-Op: 3-A, 2-B, 2-C | Required total knee arthroplasty (n=1)  Patellofemoral pain and recurrent instability (n=1) |
| Mariani et al. ^45^ (1997) | 26.5 months | Lysholm  Pre-Op: 56 +- 12  Post-Op: 94 +- 8  Tegner  Pre-Injury: 7.4  Pre-Op: 3.4  Post-Op: 5.4  IKDC  Pre-Op: All D  Post-Op: 6-A, 13-B, 3-C, 2-D  Posterior displacement (arthrometer)  Pre-Op: 10.79 +- 2.15 mm  Post-Op: 6.5 +- 3.19 mm  Side to side difference (arthrometer)  Pre-Op: 8.38 +- 1.95 mm  Post-Op: 4.08 +- 2.09 mm  Posterior Drawer  Post-Op: 6 normal, 13 Grade 1, 3 Grade 2, 2 Grade 3 | None |
| Noh et al. ^48^ (2017) | 27.7 months | Lysholm  Pre-Op: 58 +- 9  Post-Op: 91 +- 5  Tegner  Pre-Injury: 7  Post-Op: 6  IKDC  Post-Op: 17-A, 8-B, 3-C  Side to side differences (Telos)  Pre-Op: 10.4 +- 2.8 mm  Post-Op: 2.3 +- 1.8 mm  Posterior Drawer  Post-Op: 22 Grade 0, 3 Grade 1, 3 Grade 2. | None |
| Norbakhsh et al.^49^ (2014) | 42 months (36-46) | Lysholm  Pre-Op: 59 +- 10  Post-Op: 90 +- 7  IKDC  Pre-Op: All patients C/D  Post-Op: 42-A/B, 10-C/D  Side to side difference (arthrometer)  Pre-Op: 12 +- 3.9 mm  Post-Op: 3.8 +- 2.3 mm  Posterior Drawer  Pre-Op: 36 Grade 3, 16 Grade 4  Post-Op: 41 Grade 1, 9 Grade 2, 2 Grade 3 | None |
| Ochiai et al. ^50^ (2018) | 24 mo. | Lysholm  Pre-Op: 45.5  Post-Op: 79.9  Tibial translation (Telos)  Pre-Op: 8.9 mm  Post-Op: 4.2 mm | None |
| Rauck et al. ^55^ (2019) | 6.3y (1.4-15.2) | Lysholm  Post-Op: 83.1 +- 17.9  Tegner  Post-Op: 6.13 +- 2.61  IKDC  Post-Op: 77.3 +- 16.5 | No graft failures |
| Rhatomy et al. ^56^ (2021) | 24 mo. | Lysholm  HT Group Pre-Op: 53.8 +- 10.8  Post-Op: 84.2 +- 5.0  PLT Group Pre-Op: 50.07 +- 10.00  Post-Op: 83.2 +- 5.8  IKDC  HT Group Pre-Op: 52.8 +- 10  Post-Op: 82.5 +- 4.9  PLT Group Pre-Op: 49.8 +- 10.3  Post-Op: 81.3 +- 5.8 | Anterior knee pain (n=6)  Kneeling pain (n=6)  Posterior thigh pain (n=6)  Paresthesia in infrapatellar area (n=4) |
| Saragaglia et al. ^58^ (2020) | 24 mo. | Lysholm  HT Group Pre-Op: 61.3 +- 7.6  Post-Op: 85.3 +- 5.1  LARS Group Pre-Op: 63.4 +- 5.7  Post-Op: 86.7 +- 4.5  Tegner  HT Group Pre-Op: 3.1 +- 0.6  Post-Op: 7.1 +- 0.8  LARS Group Pre-Op: 3.1 +- 0.6  Post-Op: 7.2 +- 0.7  IKDC  HT Group Pre-Op: 58.3 +- 6.3  Post-Op: 83.6 +- 5.7  LARS Group Pre-Op: 60.1 +- 5.4  Post-Op: 81.9 +- 4.6  Posterior laxity  HT Group Pre-Op: 18mm  Post-Op: 7.37 mm  LARS Group Pre-Op: 18.75mm  Post-Op: 5.25 mm | NR |
| Sekiya et al. ^62^ (2005) | 5.9y (2.6-11) | IKDC  Post-Op: 9-A, 3-B, 7-C, 2-D  Side-to-side difference  Post-Op: 1.96 mm  Posterior Drawer  Post-Op: 50% nearly normal, 50% abnormal | NR |
| Seon et al. ^63^ (2006) | Group A: 31.8 (24-58)  Group B: 35.9 (24-80) | Lysholm  Group A Pre-Op: 55.5  Post-Op: 91.3  Group B Pre-Op: 52.2  Post-Op: 92.8  Tegner  Group A Pre-Op: 2.6  Post-Op: 5.6  Group B Pre-Op: 2.9  Post-Op: 6.1  Posterior Drawer  Group A Pre-Op: 5 Grade 2, 16 Grade 3  Post-Op: 19 Grade 1, 2 Grade 2  Group B Pre-Op: 7 Grade 2, 15 Grade 3  Post-Op: 20 Grade 1, 2 Grade 2  Side-to-side differences (Telos)  Group A Pre-Op: 12.3 +- 2.1 mm  Post-Op: 3.7 +- 2.1 mm  Group B Pre-Op: 11.0 +- 1.7 mm  Post-Op: 3.3 +- 1.6 mm | Anterior knee pain (n=1)  Crepitus (n=12)  Paresthesia (n=6)  Pain around patella (n=4) |
| Shon et al. ^65^ (2010) | Group A: 90.5 (71-101)  Group B: 62 (55-76) | Lysholm  Group A Pre-Op: 43.3 +- 7.04  Post-Op: 88.1 +- 7.32  Group B Pre-Op: 44.7 +- 5.02  Post-Op: 88.7 +- 9.11  Tegner  Group A Pre-Op: 2.9 +- 1.02  Post-Op: 7.4 +- 1.08  Group B Pre-Op: 2.7 +- 1.01  Post-Op: 8.1 +- 0.93  Posterior Drawer  Group A Pre-Op: 11 Grade 2, 3 Grade 3  Post-Op: 13 Grade 1, 1 Grade 2  Group B Pre-Op: 11 Grade 2, 5 Grade 3  Post-Op: 15 Grade 1, 1 Grade 2  Side-to-side differences (Telos)  Group A Pre-Op: 9.8 +- 1.7 mm  Post-Op: 3.0 +- 1.1 mm  Group B Pre-Op: 10.7 +- 1.6 mm  Post-Op: 2.6 +- 0.49 mm | 10 degree knee flexion limitation compared to contralateral knee (n=3)  Quadriceps atrophy (n=3)  Pain around staple (n=2) |
| Song et al. ^66^ (2014) | TT Group: 139 months  TI Group: 144 months | Lysholm  TT Group Pre-Op: 59.9 +- 16.4  Post-Op: 89.9 +- 9.7  TI Group Pre-Op: 54.5 +- 13.8  Post-Op: 92.1 +- 10.4  Tegner  TT Group Pre-Op: 2.5  Post-Op: 5.9  TI Group Pre-Op: 2.3  Post-Op: 6.0  Side to side difference (Telos)  TT Group Pre-Op: 10.1 mm  Post-Op: 4.1 mm  TI Group Pre-Op: 10.4 mm  Post-Op: 4.2 mm | Saphenous nerve distribution numbness + mild tenderness at harvesting site (n=5)  Scar discomfort (n=3) |
| Tachibana et al. ^69^ (2021) | 14 months | Posterior Tibial Translation (radiograph)  Pre-Op: 10.0 +- 3.6 mm  Post-Op: 3.6 +- 2.1 mm  Posterior Drawer Test  Pre-Op: All patients Grade 2/3  Post-Op: All patients Grade 1/2 | NR |
| Wang et al. ^72^ (2004) | Autograft Group: 33 months  Allograft Group: 34 months | Lysholm  Autograft Post-Op: 87.8 +- 9.6  Allograft Post-Op: 92.3 +- 6.8  Tegner  Autograft Post-Op: 4.73 +- 1.66  Allograft Post-Op: 4.7 +- 1.66  IKDC  Autograft Post-Op: 11-A, 12-B, 5-C, 4-D  Allograft Post-Op: 5-A, 9-B, 5-C, 4-D  Side to side difference (arthrometer)  Autograft Post-Op: 3.16 +- 2.60  Allograft Post-Op: 2.83 +- 1.70 | Infections (n=2)  Donor Site pain (n=4)  Reflex sympathetic dystrophy (n=1) |
| Wong et al. ^76^ (2009) | 45-48 months | Lysholm  AM group Pre-Op: 52 +- 22  Post-Op: 88 +- 10  AL group Pre-Op: 50 +- 16  Post-Op: 91 +- 8  Tegner  AM group Pre-Op: 2.3 +- 1.6  Post-Op: 4.4 +- 1.6  AL group Pre-Op: 2.2 +- 1.6  Post-Op: 5.1 +- 1.7  IKDC  AM group Pre-Op: 51.4 +- 13.3  Post-Op: 73.3 +- 11.2  AL group Pre-Op: 48.1 +- 12.4  Post-Op: 74.1 +- 11.7  Side to side difference (arthrometer)  AM group Pre-Op: 5.8 +- 2.6  Post-Op: 2.8 +- 1.6  AL group Pre-Op: 6.3 +- 2.8  Post-Op: 3.3 +- 2.8 | NR |
| Wu et al. ^77^ (2007) | 66 (60-76) | Lysholm  Pre-Op: 67 +- 9  Post-Op: 89 +- 10  Tegner  Pre-Injury: 7.2 +- 1.4  Pre-Op: 3 +- 1.7  Post-Op: 6.0 +- 2.6  IKDC  Pre-Op: 5-B, 7-C, 10-D  Post-Op: 5-A, 13-B, 4-C  Side to side difference (arthrometer)  Pre-Op: 11 +- 3.6 mm  Post-Op: 3.5 +- 2.7 mm  Posterior Drawer  Pre-Op: 14 Grade 3, 8 Grade 4  Post-Op: 16 Grade 1, 4 Grade 2, 2 Grade 3 | ROM restriction (n=1)  Hardware removal at tibial fixation(n=2) |
| Xu et al. ^78^ (2014) | 51 months (46-57) | Lysholm  HS Group Pre-Op: 56.2 +- 7.7  Post-Op: 87.9 +- 7.7  LARS Group Pre-Op: 68.4 +- 9.8  Post-Op: 87.0 +- 6.8  Tegner  HS Group Pre-Op: 3.38 +- 0.89  Post-Op: 6.31 +- 0.79  LARS Group Pre-Op: 3.21 +- 0.63  Post-Op: 6.42 +- 0.84  IKDC  HS Group Pre-Op: 13-C, 3-D  Post-Op: 9-A, 6-B, 1-C  LARS Group Pre-Op: 12-C, 7-D  Post-Op: 10-A, 7-B, 2-C  Posterior displacement (Arthrometer)  HS Group Pre-Op: 14.03 +- 1.82 mm  Post-Op: 3.28 +- 1.95 mm  LARS Group Pre-Op: 13.68 +- 1.49 mm  Post-Op: 3.27 +- 2.13 mm | Anteriomedial knee pain (n=2)  Residual paresthesia on medial thigh (n=3)  Severe synovitis (n=1) |
| Yang et al. ^79^ (2012) | Group A: 60  Group B: 58 | Lysholm:  Pre-Op: 47 +- 7  Post-Op: 89 +- 5  Tegner  Pre-Injury: 7 +- 1.2  Pre-Op: 2.2 +- 1.3  Post-Op: 6.0 +- 1.2  IKDC  Pre-Injury: 37-A, 21-B  Pre-Op: 12-C, 46-D  Post-Op: 19-A, 31-B, 7-C, 1-D  Side to side difference (Telos)  Pre-Op: 12.6 +- 1.4 mm  Post-Op: 3.5 +- 2.1 mm | Bony fracture (n=2)  Detachment between bone and tendinous portion (n=2) |
| Yoon et al. ^80^ (2019) | SB Group: 10.4y  DB Group: 10.9y | Lysholm  SB Group Post-Op: 73.8 +- 21.5  DB Group Post-Op: 74.4 +- 18.4  Tegner  SB Group Post-Op: 5.0 +- 1.8  DB Group Post-Op: 4.8 +- 1.8  IKDC  SB Group Post-Op: 67.2 +- 22.5  DB Group Post-Op: 67.5 +- 20.7  Side to side differences (Telos)  SB Group Pre-Op: 10.2 +- 3.4 mm  Post-Op: 5.3 +- 3.5 mm  DB Group Pre-Op: 9.8 +- 4.3 mm  Post-Op: 5.0 +- 3.8 mm | Graft failure (n=10)  Osteoarthritis progression (n=9) |
| Zayni et al. ^82^ (2011) | 29 (12-48) | Lysholm  Pre-Op: 41.5 +- 18  Post-Op: 81.7 +- 10  Tegner  Pre-Injury: 7.8 +- 1.7  Pre-Op: 2.8 +- 2.2  Post-Op: 6.7 +- 2  IKDC  Pre-Op: 39.5 +- 21; 10-C, 11-D  Post-Op: 74.5 +- 17.7; 8-A, 9-B, 4-C  Posterior Drawer  Pre-Op: 11.9 +- 3.3 mm  Post-Op: 3.6 +- 2.2 mm | Reflex sympathetic dystrophy syndrome (n=3)  Transitory mild knee pain (n=4) |
| Zhao et al. ^83^ (2007) | 4SHG Group: 2.6 years  7SHG Group: 2.5 years | Lysholm  4SHG Post-Op: 83 +- 4  7SHG Post-Op: 92 +- 4  IKDC  4SHG Post-Op: 11-A, 5-B, 4-C, 1-D  7SHG Post-Op: 14-A, 6-B, 2-C  Side to side differences (arthrometer)  4SHG Post-Op: 3.7 +- 1.6 mm  7SHG Post-Op: 1.7 +- 1.4 mm  Posterior Drawer  4SHG Pre-Op: 18 Grade 2, 3 Grade 3  Post-Op: 11 Grade 1, 9 Grade 2, 1 Grade 3  7SHG Pre-Op: 18 Grade 2, 4 Grade 3  Post-Op: 15 Grade 1, 7 Grade 2. | Uncomfortable on medial side of knee at full flexion (n=5)  Infection (n=1) |
| Zhao et al. ^84^ (2008) | Min. 24 months | Lysholm  Pre-Op: 58.6 +- 4.4  Post-Op: 94.9 +- 3.6  Tegner  Pre-Injury: 7.1  Pre-Op: 5.6  Post-Op: 6.9  IKDC  Pre-Op: 64.1 +- 3.3; All C/D  Post-Op: 95.6 +- 3.1; 16-A, 2-B  Side-to-side difference (arthrometer)  Pre-Op: 9.3 +- 1.4 mm  Post-Op: 0.7 +- 0.9 mm  Posterior Drawer  Pre-Op: 13 Grade 2, 5 Grade 3  Post-Op: 17 negative, 1 Grade 1 | None |
| Zhao et al. ^85^ (2009) | Min. 24 months | Lysholm  MSA Group Pre-Op: 45.3 +- 6.6  Post-Op: 95.0 +- 4.6  LSA Group Pre-Op: 44.7 +- 8.1  Post-Op: 93.7 +- 4.2  Tegner  MSA Group Pre-Op: 3.9 +- 0.9  Post-Op: 5.4 +- 0.9  LSA Group Pre-Op: 4.1 +- 0.8  Post-Op: 5.6 +- 0.7  IKDC  MSA Group Pre-Op: 54.6 +- 8.8  Post-Op: 93.1 +- 3.8  LSA Group Pre-Op: 52.9 +- 8.9  Post-Op: 92.6 +- 4.1  Average laxity (arthrometer)  MSA Group Pre-Op: 9.7 +- 3.5  Post-Op: 1.6 +- 1.2  LSA Group Pre-Op: 9.9 +- 4.3  Post-Op: 1.5 +- 1.3 | None |

Legend: IKDC, International Knee Documentation Committee; NR, not reported; QT, quadriceps tendon; HT, hamstring tendon; PLT, peroneus longus tendon; SB, single bundle; DB, double bundle; 4S, four strand; 2S, two strand; 7S, seven strand; ROM, range of motion; PT, patellar tendon; TA, tibialis anterior; LARS, ligament augmentation and reconstruction system; AM, anteromedial; AL, anterolateral; 4SHG, 4-strand hamstring graft; 7SHG, 7-strand hamstring graft; MSA, medial-sided augmentation; LSA, lateral-sided augmentation; TT, transtibial; TI, tibial inlay; Min., minimum; Pre-Op, preoperative; Post-Op, postoperative; mm, millimeters.
